# Ribitol enhances matriglycan of α-dystroglycan in breast cancer cells without affecting cell growth

**DOI:** 10.1038/s41598-020-61747-z

**Published:** 2020-03-18

**Authors:** Pei J. Lu, Jason D. Tucker, Elizabeth K. Branch, Fei Guo, Anthony R. Blaeser, Qi L. Lu

**Affiliations:** 1 McColl-Lockwood Laboratory for Muscular Dystrophy Research, Atrium Health, Charlotte, NC 28203 USA; 2 Immune Monitoring Core, Atrium Health, Charlotte, NC 28203 USA

**Keywords:** Cancer, Cell biology, Drug discovery, Molecular biology, Diseases, Medical research, Molecular medicine, Oncology

## Abstract

The laminin-binding glycan (matriglycan) on α-dystroglycan (α-DG) enables diverse roles, from neuronal development to muscle integrity. Reduction or loss of matriglycan has also been implicated in cancer development and metastasis, and specifically associated with high-grade tumors and poor prognoses in breast cancers. Hyperglycosylation of α-DG with LARGE overexpression is shown to inhibit cancer cell growth and tumorigenicity. We recently demonstrated that ribitol, considered to be a metabolic end-product, enhances matriglycan expression in dystrophic muscles *in vivo*. In the current study, we tested the hypothesis that ribitol could also enhance matriglycan expression in cancer cells. Our results showed for the first time that ribitol is able to significantly enhance the expression of matriglycan on α-DG in breast cancer cells. The ribitol effect is associated with an increase in levels of CDP-ribitol, the substrate for the ribitol-5-phosphate transferases FKRP and FKTN. Direct use of CDP-ribitol is also effective for matriglycan expression. Ribitol treatment does not alter the expression of *FKRP, FKTN* as well as *LARGEs* and *ISPD* which are critical for the synthesis of matriglycan. The results suggest that alteration in substrates could also be involved in regulation of matriglycan expression. Interestingly, expression of matriglycan is related to cell cycle progression with highest levels in S and G2 phases and ribitol treatment does not alter the pattern. Although matriglycan up-regulation does not affect cell cycle progression and proliferation of the cancer cells tested, the novel substrate-mediated treatment opens a new approach easily applicable to experimental systems *in vivo* for further exploitation of matriglycan expression in cancer progression and for therapeutic potential.

## Introduction

Dystroglycan (DG) is a major adhesion molecule highly conserved in mammals^1–4^. DG is transcribed as a single transcript, and post- translationally cleaved into two subunits, α-DG and β−DG. The transmembrane and cytoplasmic subunit, β-DG, binds to cytoplasmic proteins, whereas α-DG, the extracellular subunit, interacts with laminin-G domain-containing extracellular matrix (ECM) proteins including laminin, agrin and perlecan^5^. This interaction is mediated through the laminin-binding glycan (matriglycan) of α-DG (F-α-DG)^6^. Matriglycan-modified α-DG is expressed in most tissues and plays diverse roles, from acting as viral receptors to neuronal development^7,8^. After nearly 3 decades of intensive pursuit to understand the pathway leading to the synthesis of matriglycan of α-DG, significant progress has been made with the structure of the glycan chain on the core M3 of α-DG delineated:

(3GlcA-1-3Xyl-1)*n*-3GlcA-1-4Xyl-Rbo5P-1Rbo5P-3GalNAc-1-3GlcNAc-1-4(P-6)Man-1-Thr/ser^9–12^. The extension of the glycan chain is completed by the following proposed transferase activity sequentially: POMT1 and POMT2 catalyze the initial *O-*mannosylation of the protein^13^, and further extension of the sugar chain is executed by POMGnT2 (GTDC2)^14,15^ and B3GALNT2^16^, FKTN^9,17,18^, FKRP^9,17,18^, TMEM5^12^ and B4GAT1 successively^11^. Finally, LARGE, a bifunctional glycosyltransferase, adds the laminin-binding repeating units of 3GlcA-1-3Xyl-1^19^.

Dystroglycan is expressed in all known epithelial tissues from prostate and mammary glands to squamous epithelium, with the matriglycan-modified α-DG localized most prominently to the basal domain of epithelium in contact with ECM of the basement membrane^20–22^. However, despite specific patterns of localization, the roles of matriglycan in the development, maintenance, repair and functions of epithelial tissues remain largely elusive. Lack or reduced expression of matriglycan due to mutations in *LARGE*, *FKRP* and other glycosyltransferases shows limited effect on epithelial structure and functions in postnatal humans and animals^22–24^. However, several lines of evidence suggest that altered expression and distribution of matriglycan may contribute to cancer development, progression and metastasis^24–26^. First, matriglycan is reduced or lost in a variety of human primary cancer cells and cell lines, including prostate, breast and colorectal cancers^20,21,23,27^. Second, the most pronounced reduction in expression levels of matriglycan is often observed in high-grade tumors with high proliferation index, and not surprisingly appears to be correlated with poor prognosis^24,25,28,29^. In most primary tumors and in cancer cell lines, the levels of DG protein expression are relatively constant, indicating that it is the glycosylation rather than the DG expression which is altered in the process of tumorigenesis and progression. Finally, exogenous expression of LARGE via virus-mediated gene transfer can achieve significant inhibition of cancer cell proliferation^30–32^. Since LARGE overexpression only increases matriglycan, but not DG protein expression, the result therefore further supports the hypothesis that alteration in the laminin-binding glycan of α-DG plays a role in cancer development and progression, and that increasing expression of matriglycan could be a novel therapeutic approach for cancers.

Recently, the pentose alcohol ribitol has been reported with the capacity to enhance the production of CDP-ribitol in muscle tissues and restore the expression of matriglycan in a dystroglycanopathy mouse model with FKRP mutations. This led to significant improvement in muscle pathology and function^18,33^. This effect was not associated with alteration in FKRP and LARGE expression, therefore suggesting a new pathway of metabolite-mediated modulation of matriglycan. We hypothesized that this modulation could also occur in other cell types. Here we have examined ribitol in several human cancer cell lines and demonstrated that ribitol significantly and dose dependently enhances matriglycan production in the breast cancer cell line MCF7. Limited increase of matriglycan was also observed in the breast cancer cells T47D even though the cells already expressed high levels of matriglycan. Ribitol treatment increased the levels of CDP-ribitol, the substrate for FKRP/FKTN, but did not alter the expression of *FKRP*, *FKTN*, *LARGEs* and *ISPD* known to be essential for the synthesis of matriglycan. Importantly, treatment with CDP-ribitol enhanced matriglycan expression with higher dose efficacy than ribitol. Interestingly, levels of matriglycan was found to be related to cell cycle progression, and ribitol-enhanced matriglycan did not inhibit growth of the cancer cells. Our data adds further complexity to the regulation of matriglycan expression in cancer cells.

## Results

### Ribitol enhances expression of matriglycan of α-DG in the MCF7 breast cancer cell line

**Figure 1:**
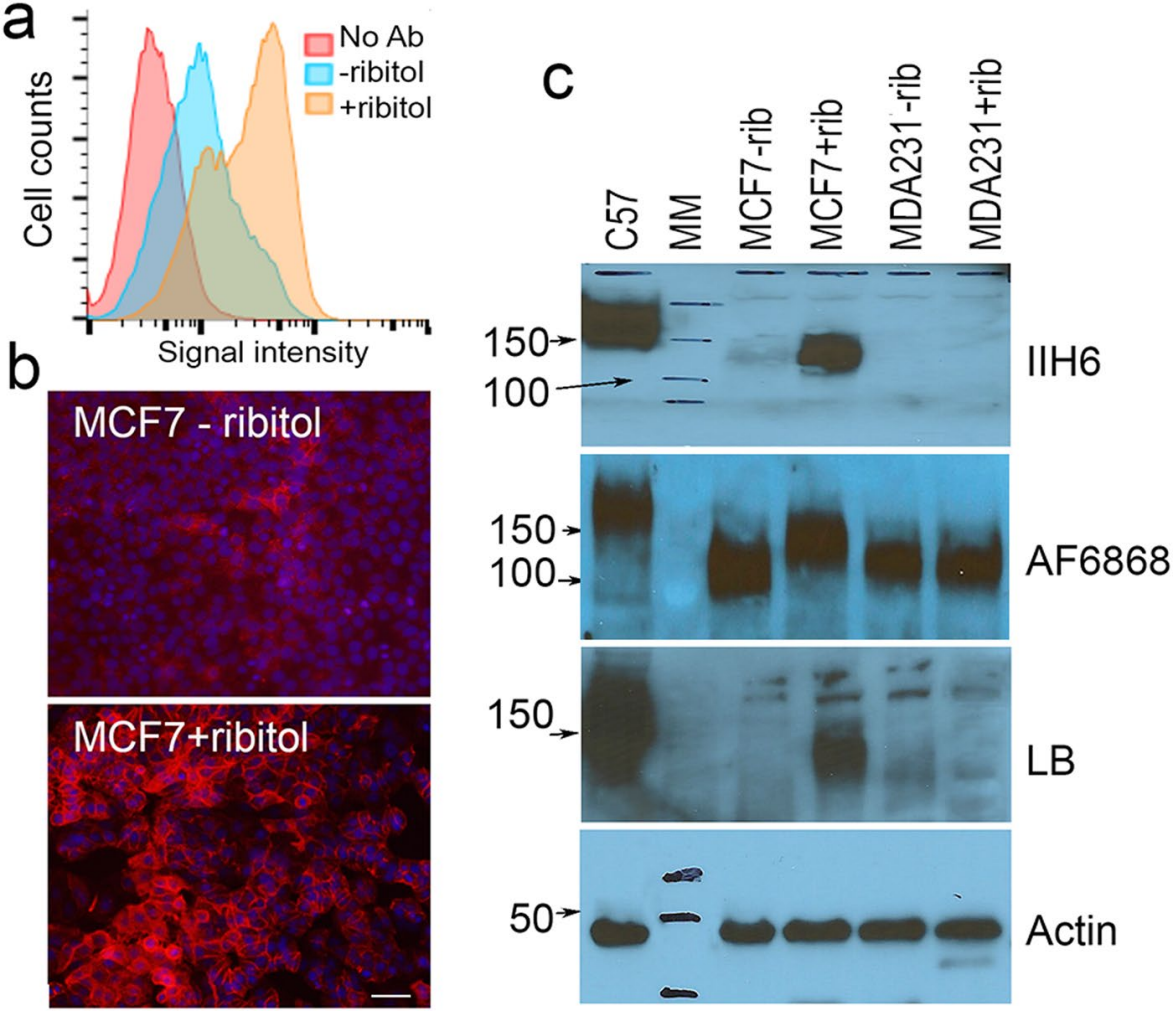
Analysis of matriglycan expression in MCF7 cells treated with 10 mM ribitol. Matriglycan is detected with IIH6 antibody by FACS (**a**) and immunocytochemistry (**b**). No Ab, untreated cells stained without primary antibody; + and − ribitol, cells treated with and without ribitol respectively and detected with IIH6. (**c**) Alpha DG is detected with antibody IIH6, AF6868 and laminin-overlay (LB). Antibody AF6868 detects core and matriglycan modified α-DG and shows increase in signal intensity of the higher molecular weight band and decrease in lower molecular weight band in the ribitol-treated MCF7 cells (+rib) when compared to the untreated cells (−rib). Stronger signal for laminin binding is detected in the ribitol-treated MCF7 cells over the untreated cells. Matriglycan is not detected in both treated and untreated MDA231 cells. C57, lysate from C57 skeletal muscle is used as positive control. MM, Lane of molecular weight marker. Scale bar, 50 μM.

We initially examined six human cancer cell lines including the breast cancer cell lines, MCF7 and MDA231; prostate cancer cell lines, LNCaP and PC3; cervical cancer Hela and metastatic lung cancer H1299 cell line. The cells were treated with ribitol at 10 mM concentration one day after passage and analyzed 3 days later for levels of matriglycan by FACS with IIH6 antibody specifically recognizing matriglycan of α-DG. There was no clear difference in signal intensity between the ribitol-treated and control cells (without ribitol supplementation) in all the cell lines except MCF7 (Fig. 1a and Supplementary Information Fig. 1a). Signal intensity was greatly enhanced in the MCF7 cells treated with ribitol when compared to the untreated control. We further assessed the variation in matriglycan expression within the cell population by immunocytochemistry (ICC) with cells cultured under the same preparation and ribitol treatment as described above. As illustrated in Fig. 1b, positive membrane signals with IIH6 were barely detectable in the majority of the untreated cells, but a small proportion of cells, especially those closely packed with small diameter, were clearly matriglycan positive. Weak signals were also detected in some cells in more confluent areas. In the ribitol-treated culture, however, expression of matriglycan was demonstrated in the majority of cells with a clear membrane localization. The strongest expression was mainly in two groups of cells, the tightly packed cells and those of larger size with polygonal shape, both of which being predominantly located in large confluent areas. Staining with AF6868 polyclonal antibody which recognizes the core of α-DG with and without matriglycan modification showed mainly membrane localized signals. No clear difference in signal intensity and distribution was observed between the treated and control groups (Supplementary Information Fig. 1b).

Enhanced expression of matriglycan by ribitol was confirmed in the MCF7 cells by western blot (WB) with the IIH6 antibody, showing a positive band with molecular weight consistent with matriglycan modified α-DG. Further evidence for ribitol effect on modification of α-DG came from WB with AF6868. A clear shift in sizes of the α-DG, from lower MW species representing α-DG core (without matriglycan) to higher MW species (matriglycan modified) recognized by IIH6, was observed in the ribitol treated cells as compared to the control. As expected, the glycosylated α-DG enhanced by ribitol was capable of laminin binding as demonstrated by the laminin binding assay. Consistently, no clear difference in matriglycan expression was observed in the other cell lines tested between ribitol treated and control as illustrated by the MDA231 (Fig. 1c).

### Dose dependent enhancement of matriglycan with ribitol treatment in MCF7 cells

**Figure 2:**
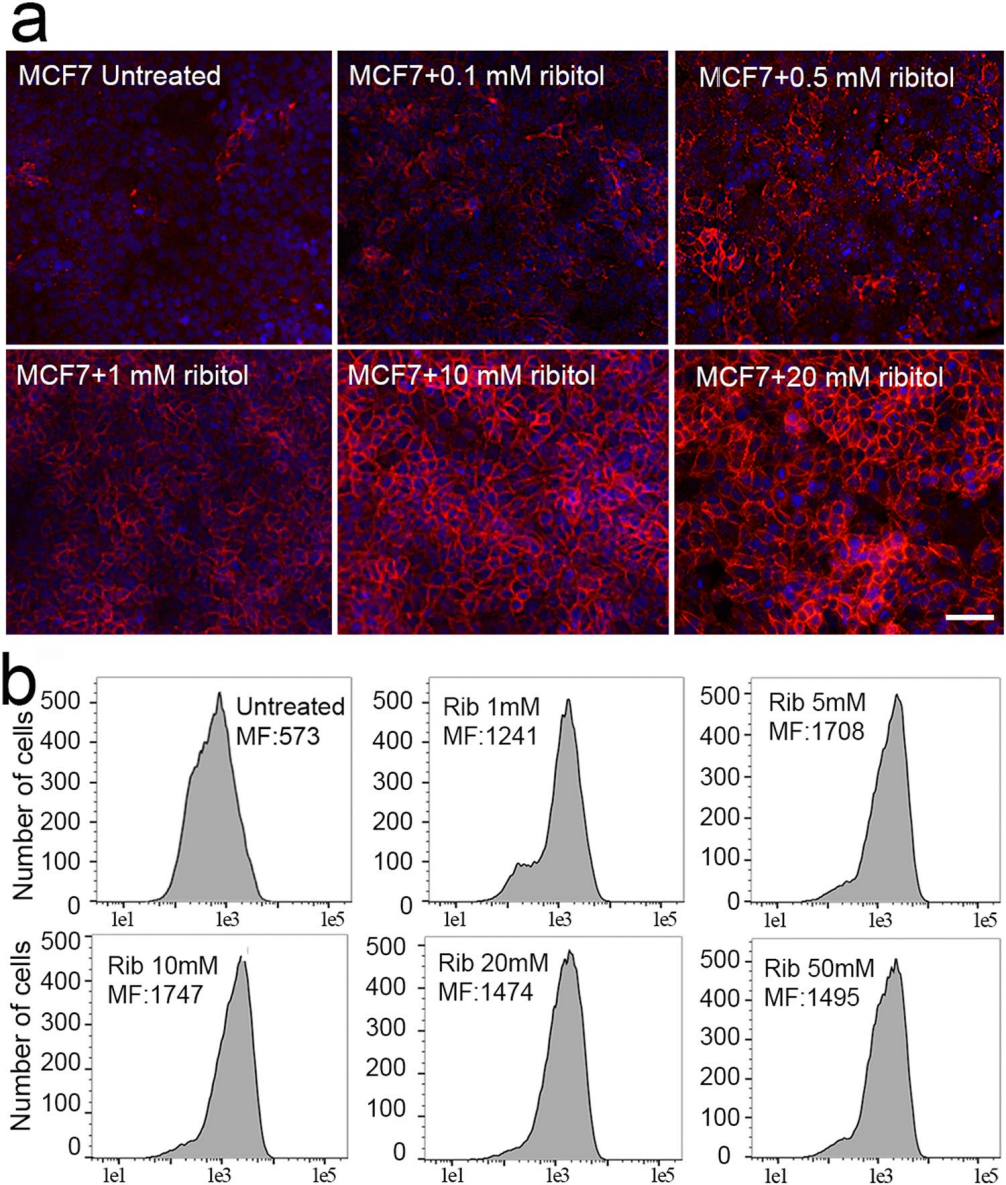
Dose response of MCF7 cells to ribitol treatment. (**a**) Detection of matriglycan with the antibody IIH6 by immunocytochemistry (scale bar, 50 μM). Blue color, nuclear staining with DAPI. (**b**) FACS analysis with antibody IIH6. Untreated cells stained with the same antibody. Rib, ribitol. MF, median fluorescence signal intensity (a.u.).

We next examined the dose effect of ribitol from 20 μM to 50 mM on the expression of matriglycan in the MCF7 cells. Under the same culture condition with 3 days of treatment, the lowest dose of 20 μM ribitol showed limited, but clearly detectable effect on levels of matriglycan. The cells treated with 0.1 mM ribitol showed a sharp increase in the number of matriglycan positive cells by ICC, from less than 5% to more than 30%, although most other cells were only weakly stained (Fig. 2a). Ribitol treatment at higher concentration increased the IIH6 positive cells further with stronger membrane signal. The majority of cells became clearly IIH6 positive at 1 mM ribitol although variation in intensity remained. Ribitol treatment at 5 mM and 10 mM resulted in strongest signal in more than 80% of the cell population with the remaining cells stained weakly (Fig. 2a). Increasing ribitol concentration in culture to 20 mM and 50 mM did not further increase the signal intensity and alter the pattern of signal distribution. The dose dependent increase in matriglycan levels was also demonstrated by FACS analysis with the same antibody (Fig. 2b). A dramatic increase in the median fluorescence signal intensity (MF:1241) was observed after ribitol treatment at 1 mM when compared to the levels (MF:573) in the control cells. Median fluorescence level was further increased at 5 mM (MF:1708) and reached a maximum (MF:1747) at 10 mM concentration. Consistent with results from ICC, signal intensity did not increase further at ribitol concentration of 20 mM and 50 mM when compared to 10 mM ribitol treated cells. As a control, we also examined a similar dose range of D-glucose with the same culture conditions and detected no clear enhancement in the expression of matriglycan by FACS at any dose (Supplementary Information Fig. 2).

### Ribitol enhances matriglycan levels in T47D cells

**Figure 3:**
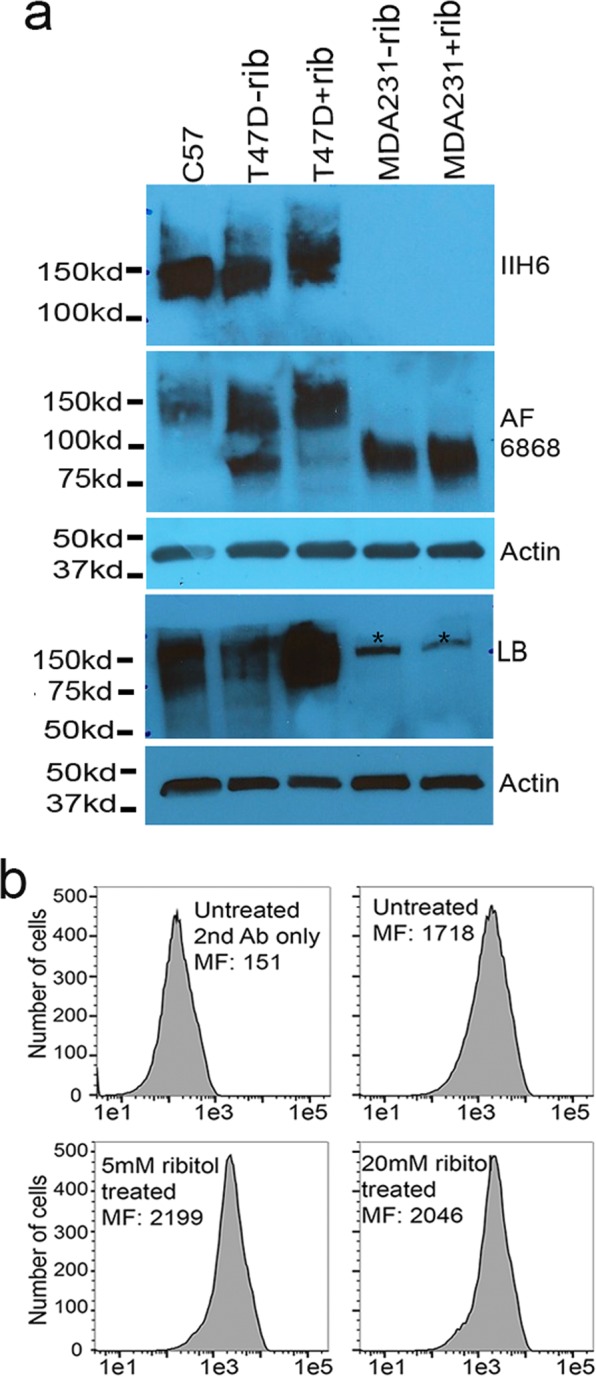
Detection of matriglycan in T47D breast cancer cells after ribitol treatment by Western blot (**a**) and FACS analysis (**b**). (**a**) C57, tissue lysate from C57 skeletal muscle used as positive control. −rib, without ribitol treatment; +rib, 5 mM ribitol treatment. MDA231 cells were used as negative control which shows signal only for core M3 glycan with and without ribitol treatment (detected by AF6868). LB, laminin binding assay. * indicate the nonspecific signal to laminin. (**b**) 2^nd^ Ab only, the cells were stained without primary IIH6 antibody.

To test whether matriglycan expression can be further enhanced in cancer cells already demonstrating strong expression of matriglycan, we examined another breast ductal carcinoma cell line T47D which has a biochemical phenotype similar to MCF7 cells, expressing receptors for estrogen, androgen and progesterone. As reported earlier, matriglycan is highly expressed in the cells and can be readily detected by western blot^34^. We treated T47D cells with 5 mM ribitol for 3 days and examined matriglycan levels using IIH6 antibody by western blot. As expected, expression of matriglycan was readily detected in the cells without ribitol treatment, with signal intensity being only slightly weaker than that from normal skeletal muscle under same amount of protein loading (Fig. 3a). Matriglycan levels were mildly enhanced from the cells treated with ribitol when compared to the untreated control. We also examined the glycosylation status of α-DG from the same cells with AF6868 antibody. The control T47D cells revealed two forms of α-DG, the strong upper band representing α-DG with matriglycan and a weaker lower band representing core α-DG without matriglycan modification. Interestingly, ribitol treatment enhanced the upper matriglycan band and diminished the lower core α-DG. Consistently, FACS analysis confirmed high levels of matriglycan in the control T47D cells with mean fluorescence intensity reaching levels higher than that observed in the ribitol-treated MCF7 cells (Fig. 3b). Consistent to the western blot results, 5 mM ribitol treatment further enhanced the matriglycan levels, but the increase in mean fluorescence intensity was limited (about 20%) in comparison to the untreated control. Higher doses of ribitol (up to 20 mM) did not further increase matriglycan signal (Fig. 3b).

Laminin binding assay showed that signals from the control T47D cells (without ribitol treatment) were much weaker than that from the normal muscle, even though signal intensity with IIH6 antibody was similar between the two samples (Fig. 3a). Interestingly, ribitol treatment greatly enhanced the laminin binding signal, up to 250% of that observed in the control cells by blot densitometry and this is confirmed by repeated tests. Overall, the results suggest that ribitol can further enhance laminin-binding matriglycan in cells already expressing abundant matriglycan detected by IIH6 when core M3 is available.

### Ribitol treatment increases cellular levels of ribitol-5-Phosphate and CDP-ribitol

**Figure 4:**
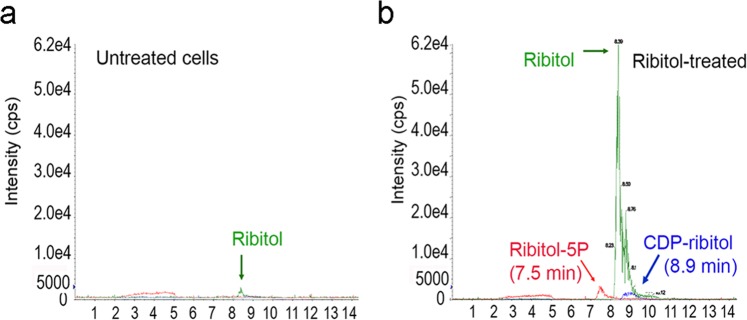
LC-MS/MS chromatogram for the detection of ribitol, ribitol-5-phosphate (ribitol-5P) and CDP-ribitol in the untreated (**a**) and 10 mM ribitol treated MCF7 cells (**b**). Quantitation of the metabolites is listed in Supplementary Table 1.

We reported earlier that externally supplied ribitol in muscle cells *in vivo* can be converted to CDP-ribitol, the substrate of FKRP and FKTN^33^. To directly assess the delivery of ribitol to epithelial cells and whether conversion of ribitol to CDP-ribitol occurs in the cells, we treated MCF7 cells with 10 mM ribitol and analyzed cellular levels of both ribitol- 5 phosphate (rib-5P) and CDP-ribitol together with ribitol by LC/MS/MS. Levels of each metabolite were determined with reference to the standard curves established with the synthesized metabolites (Supplementary Information Fig. 3). Ribitol levels in the control cells were only 6 ng per 10^5^ cells whereas the levels in the cells treated with ribitol reached 40 times (up to 243 ng per 10^5^ cells) of the control cells (Supplementary Table 1). Furthermore, the levels of both rib-5P and CDP-ribitol were approximately 5 and 10 times higher in the ribitol treated cells than that in the control cells respectively (Fig. 4a.b). The results, consistent with those observed in muscle *in vivo*, demonstrate that ribitol can effectively enter epithelial cells and be converted to rib-5P and CDP-ribitol^18,33^.

### CDP-Ribitol enhances expression of matriglycan with high efficiency

**Figure 5:**
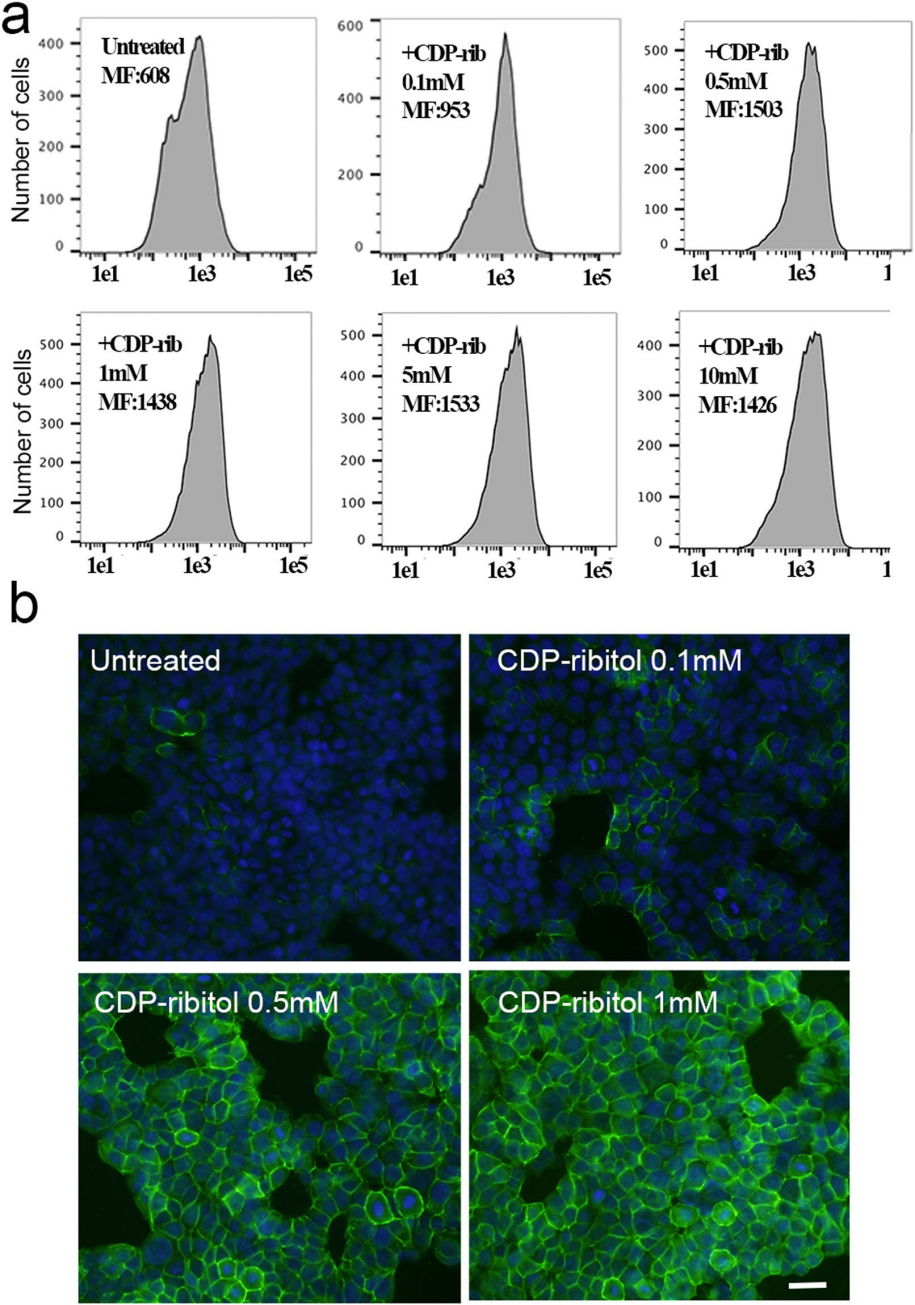
Matriglycan expression in MCF7 cells treated with CDP-ribitol and detected by FACS analysis (**a**) and immunocytochemistry (green) (**b**) with antibody IIH6. Blue nuclear staining with DAPI. +CDP-rib, CDP-ribitol; untreated MCF7 cells without ribitol treatment. MF, median fluorescence intensity. A marked increase in the expression of matriglycan is observed by both methods in the CDP-ribitol treated cells when compared to the untreated cells. Scale bar, 50 μM.

Our previous study in dystrophic mice with FKRP mutation suggests that enhanced matriglycan expression with ribitol is likely mediated through the conversion of ribitol to CDP-ribitol and the increase of the substrate for FKRP and FKTN glycosyltransferases results in a more efficient incorporation of rib-5P to existing Core M3 of α–DG, thus restoring matriglycan expression in muscles with FKRP mutation. If the same mechanism applies to the epithelial cells, direct supply of CDP-ribitol would be more effective than ribitol for enhancing glycosylation of α-DG. We therefore synthesized CDP-ribitol and examined its effect on the levels of matriglycan in the MCF7 cells. The cells were treated at 20 µM and up to 10 mM concentration of CDP-ribitol and the effect was examined by FACS with the antibody IIH6. Enhanced matriglycan was observed at all doses with median fluorescence signal (MF intensity) increased by more than 50% at 0.1 mM concentration when compared to the control (Fig. 5a). The highest level of matriglycan was detected in the cells treated with 0.5 mM to 1 mM concentration. Treatment at 5 mM and 10 mM did not further enhance the matriglycan levels beyond that observed with 1 mM treatment (Fig. 5a). The enhanced expression of matriglycan was confirmed by ICC with almost all cells strongly positive with the treatment of 0.5 mM and 1 mM concentration (Fig. 5b). Therefore, doses of CDP-ribitol required to achieve similar levels of enhancement in matriglycan are almost 10-fold lower than that of ribitol.

### Ribitol treatment does not alter the expression of *FKRP*, *FKTN*, *ISPD* and *LARGE*

**Figure 6:**
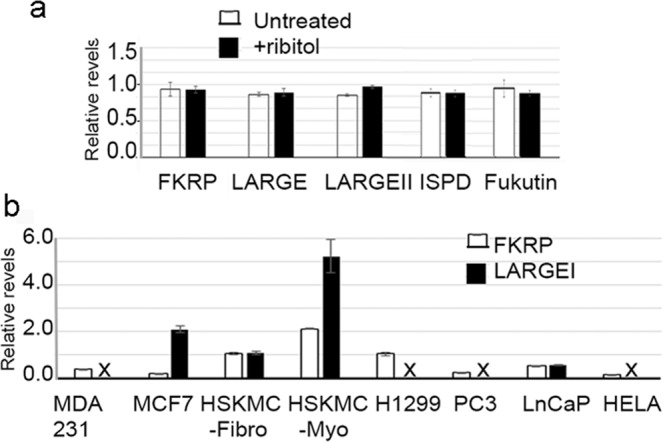
Quantitative real time RT PCR detection of *FKRP*, *LARGE*, *LARGEII*, *ISPD* and *FKTN*. (**a**) Comparison in levels of *FKRP*, *LARGE*, *LARGEII*, *ISPD* and *FKTN* between 10 mM ribitol-treated (+ribitol) and untreated MCF7 cells (n = 3, P > 0.5). (**b**) Relative levels of *LARGE* and *FKRP* expression in cell lines normalized to human skeletal fibroblast cells (HSKMC-Fibro). HSKMC-Myo, human skeletal myoblast.

The fact that both ribitol and CDP-ribitol enhance matriglycan expression suggests a possible mechanism involving expression of FKRP and FKTN. We therefore examined the mRNA levels of the two genes in the cell lines by quantitative real time PCR. However, no significant difference was detected between the treated and control MCF7 cells. We also examined the expression of *ISPD* and *LARGE* genes which have been associated with CDP-ribitol production and matriglycan synthesis of α-DG in cancer cells. Expression of *ISPD*, *LARGE, LARGEII and FKTN* showed no changes in levels of mRNA observed between the control and ribitol-treated MCF7 cells (Fig. 6a). Since the ribitol effect was most significantly observed in the MCF7 cells, but not in the other cancer cell lines, we further examined expression of *LARGE* and *FKRP* in the ribitol-non-responsive H1299, Hela, PC3 and MDA231 cells to assess potential explanation for the observed differential effect of ribitol on glycosylation of α-DG. Consistent with results reported previously (31) and in contrast with the MCF7 cells, *LARGE* expression was undetectable in H1299, Hela, and MDA231, and at very low levels in the LNCaP. Interestingly, all cell lines expressed clearly detectable levels of *FKRP* and no significant difference was observed among them (Fig. 6b). The result therefore supports the hypothesis that *LARGE* expression is also essential for ribitol-enhanced expression of matriglycan which depends on the function of FKRP and FKTN.

**Figure 7:**
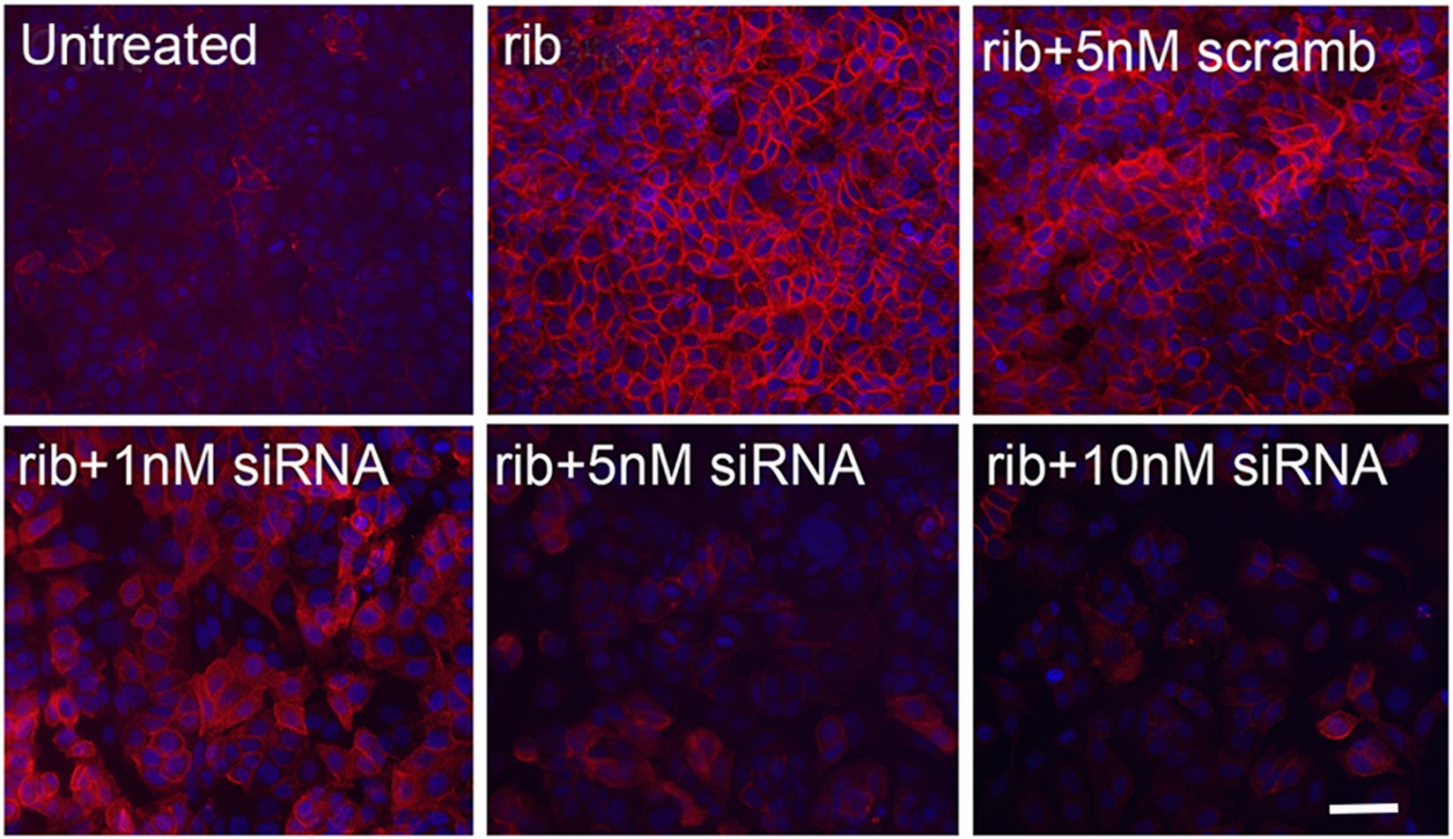
Dose dependent effect of LARGE knock down by siRNA on matriglycan expression of MCF7 cells. rib, 5 mM ribitol treatment. scramb, scramble siRNA treatment. siRNA, human LARGE specific siRNAs. Matriglycan is detected with IIH6 (red) and nuclei are stained with DAPI (blue). Scale bar, 50 μM.

To directly assess whether *LARGE* expression is essential to the ribitol-induced matriglycan expression, we transfected the MCF7 cells with a cocktail of human LARGE-specific siRNAs at 3 doses by manufacturer recommended procedure. The cells were also treated with 5 mM ribitol for 3 days. Since currently LARGE protein expression cannot be detected reliably, the expression of *LARGE* mRNA was measured by qRT-PCR. Levels of *LARGE* mRNA were dose dependently reduced, down to approximately 40% and 20% of the control levels in the cells with 1 nM and 10 nM siRNA treatment respectively (Supplementary Information Fig. 4a). Consistently, immunocytochemistry with IIH6 antibody revealed a clear dose-dependent decrease in matriglycan signal when compared to the control without siRNA treatment as well as the cells treated with scrambled siRNA (Fig. 7). LARGE-specific siRNA also dose-dependently reduced the levels of *LARGE* mRNA, but with less efficiency in the T47D cells (Supplementary Information Fig. 4b). As a result, signals for matriglycan remained easily detectable although at reduced levels in the siRNA- treated cells when compared to the untreated control (Supplementary Information Fig. 4c). Interestingly, IIH6 signals were mainly diffused in cytoplasm with slightly stronger signal around the cell membrane. However, clearly membrane localized signals were only sporadically observed in a small number of cells. No change in signal localization was observed with ribitol treatment or siRNA treatment. This result suggests that alteration in distribution of matriglycan-modified α-DG might also be involved in cancer progression, while the mechanism for the altered distribution remains unclear.

### Effects of ribitol treatment on cell proliferation

**Figure 8:**
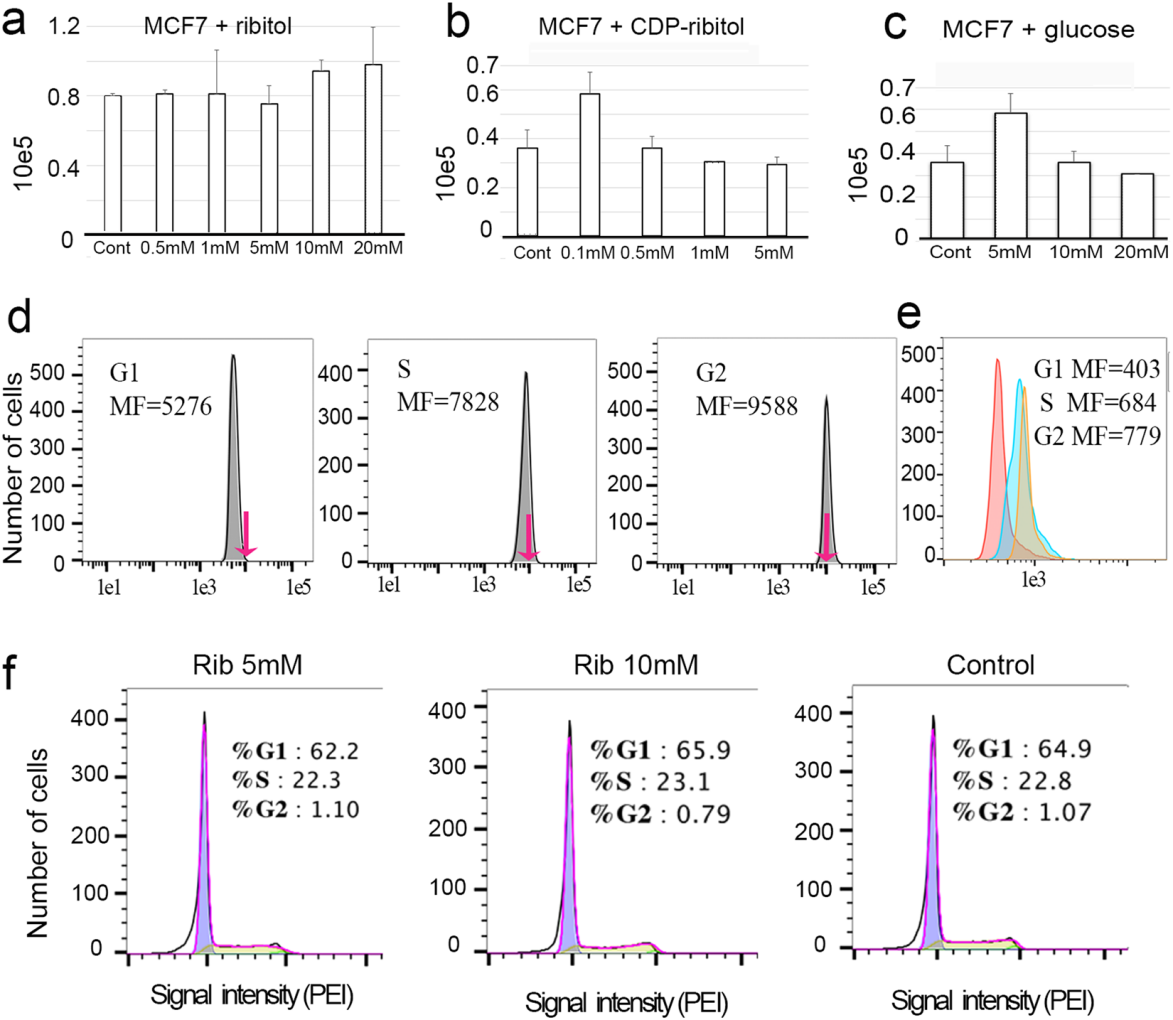
Dose effect of ribitol (**a**), CDP-ribitol (**b**) and glucose (**c**) on cell growth in MCF7 cells. Cont, untreated cells. Cells were cultured for 3 days after treatment and total number of cells were counted for comparison between treated and control (n = 3, *P* > *0.5*). FACS analysis of cell cycle progression and matriglycan expression in MCF7 cells with ribitol treatment (**d–f**). (**d**) Matriglycan expression in 5 mM ribitol treated cells at the G1, S and G2 phase with increasing signal intensity indicated by median fluorescence (MF) intensity (arrows indicate the shift in signal intensity). (**e**) Matriglycan expression in the untreated MCF7 cells at the G1, S and G2 phases. (**f**) Percentage of cells at each of the G1, S and G2 phases following 5 mM and 10 mM ribitol (Rib) treatment in comparison to the untreated cells (control).

Enhanced expression of laminin binding α-DG has been associated with inhibition in cancer cell proliferation^30,35^. We therefore examined cell growth with 0.5 mM to 20 mM ribitol treatment for 3 days in comparison with the untreated control. No significant difference in cell number was detected between the controls and the ribitol-treated cultures at any dosage. In fact, the number of cells was slightly higher in the ribitol treatment cohort with the highest doses (Fig. 8a). We also examined cell growth with CDP-ribitol at a dose range from 0.1 mM to 5 mM. Similarly to the ribitol treatment, no significant difference was detected between the CDP-ribitol treated and control cells (Fig. 8b). Since the highest efficiency in enhancing matriglycan was achieved with 5 mM ribitol and 0.5 mM CDP-ribitol, these results suggest that the enhanced expression of matriglycan does not alter the cell proliferation rate. To assess whether ribitol-induced matriglycan might take longer than 3 days to reveal any inhibitory effects on cell growth, we counted the cell number 6 days after ribitol treatment. As illustrated in Supplementary Information Fig. 5, the number of cells with 5 mM and 10 mM ribitol treatment was again slightly higher than that of the control cells.

As a control, we also examined the cell growth with D-glucose treatment at concentrations ranging from 5 mM to 20 mM. There was a slight increase in cell growth with the lowest 5 mM glucose concentration, but the doses of 10 mM and higher decreased cell growth slightly (Fig. 8c) consistent with an earlier report^36^.

### Expression of matriglycan is associated with cell cycle

To examine whether the variation in expression of matriglycan observed in the control, and particularly in the ribitol-treated cells, is cell cycle related, we analyzed the MCF7 cells using propidium iodide (PI) DNA staining in cells treated with 5 mM ribitol for 3 days. The levels of matriglycan were determined at each phase of cell cycle. Interestingly, level of matriglycan was lowest in the cells at G1 phase, considerably higher in the cells at the S phase, and the highest in the cells at the G2 phase. Similar results were obtained with repeated experiments and the same pattern was observed in the cells treated with 10 mM ribitol (Fig. 8d). A similar pattern of expression was observed in the untreated cells with the higher levels of matriglycan in the cells at S and G2 phases and the lowest at G1 phase (Fig. 8e). Furthermore, the proportion of cells at different phases of cell cycle did not show significant difference between the ribitol-treated and untreated control (Fig. 8f). The results therefore suggest that matriglycan expression is associated with cell cycle and ribitol treatment does not alter cell cycle progression in the MCF7 cells.

### Enhanced matriglycan does not inhibit cell migration or growth in Matrigel

**Figure 9:**
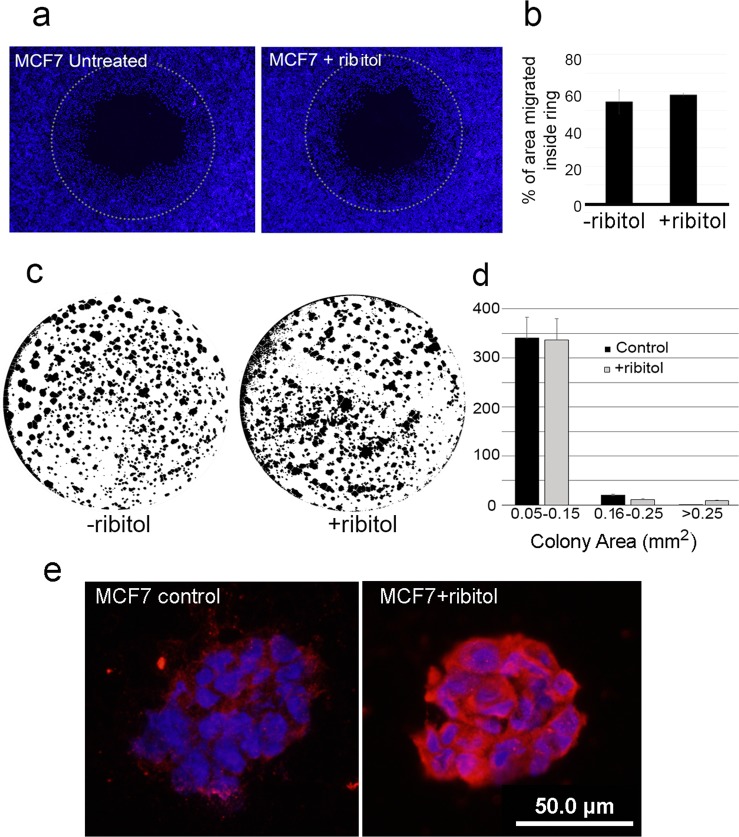
Effect of ribitol treatment on cell growth and migration. (**a**) Cell migration 4 days after the lifting of the center ring stopper (indicated by the dotted line circles). (**b**) Area size inside the center ring covered by migrated cells in both untreated (-ribitol) and 10 mM ribitol-treated (+ribitol) MCF7 cells (n = 3, *P* > 0.5). (**c**) Representative images of the MCF7 cells grow in Matrigel without (-ribitol) and with 10 mM ribitol (+ribitol). (**d**) Colony numbers and area of the MCF7 cells in Matrigel without (Control) and with 10 mM ribitol (+ribitol) treatment. Numbers in x-axis is the size of colony in mm^2^ (n = 7, *P* > 0.05). ImageJ Software (ver. 1.51 P), https://imagej.nih.gov/ij. (**e**) Examination of matriglycan expression of the MCF7 cells in the Matrigel for 7 days by immunocytochemistry with IIH6 antibody. Control, untreated cells; +ribitol, 10 mM ribitol treated cells. Blue is nuclear staining with DAPI and red with IIH6.

To examine whether ribitol treatment with enhanced matriglycan expression might alter cell behavior related to tumorigenesis, we assessed the effect of ribitol treatment on cell migration in culture plates where the middle area of the wells was separated by a ring stopper as a barrier. Once the surrounding cells reached confluence, the stopper was removed. By day 2, both treated and untreated cells have migrated into the center space with no apparent difference between the two groups. At day 4, the cells were stained with Hoechst and the size of the area covered by the migrated cells within the center ring was measured. Cells from both groups covered about 50% of the ringed area with no significant difference (Fig. 9a,b). Similar results were observed in repeated experiments. We also examined the effect of ribitol treatment on MCF7 cells grown in Matrigel which contains abundant laminins, primary ligands of the matriglycan of α-DG. The cells formed colonies with a range of sizes from single cell to those covering an area larger than 0.25mm^2^ within the Matrigel. The overall counts and the areas covered by the colonies showed no significant difference between the untreated and ribitol-treated cells (Fig. 9c,d). However, there were some differences in sizes of the colonies. Specifically, there were a few more colonies of large sizes in the ribitol-treated culture, but statistic difference was not observed. The irregular shape of these colonies suggests that they likely formed through the fusion of several smaller colonies in the vicinity. Matriglycan expression of the cells within the Matrigel for 7 days was also examined by immunocytochemistry with IIH6 antibody. As expected, only weak background staining was observed in the control cells whereas strong membrane signals were detected in the cells treated with ribitol (Fig. 9e).

## Discussion

Early studies in cancer samples from clinics have reported a correlation between hypoglycosylation of α-DG and higher tumor grades and poor prognosis in several common cancers including breast and prostate cancers. Evidence from studies *in vitro* and *in vivo* also supports the notion that hyperglycosylation of α-DG, such as that induced by *LARGE* overexpression with gene transfer approach, can achieve reduced cell proliferation, migration and tumorigenicity in cancer cell lines^31,32^. Similarly, reduction in cell migration induced by B4GAT1 (Formerly known as B3GNT1) overexpression is also considered the consequence of matriglycan upregulation^37^. These evidences have led to the hypothesis that laminin binding matriglycan is a tumor suppressor and increasing expression of matriglycan could be a new approach of cancer treatment by inhibiting or reducing cancer cells’ invasive and metastatic potential. In the current study, we demonstrated that ribitol, a little-known metabolite, is able to increase matriglycan in the breast cancer cell line MCF7, and to a limited degree in T47D cells which already expresses high levels of matriglycan. This effect is dose dependent in the MCF7 cells. The enhancement of matriglycan expression in MCF7 cells is not related to alteration in expression of *LARGE, FKRP, FKTN* and *ISPD*, known to be essential in the later steps of matriglycan synthesis on α-DG. However, *LARGE* appears essential as its expression is clearly detected in the MCF7 cells, but undetectable or barely detectable in the other ribitol non-responsive cell lines. This is consistent with the report from de Bernabe DB, *et al*. that restoration of matriglycan expression can only be achieved by overexpression of exogenous *LARGE*, but not other related glycosyltransferases in the cancer cell lines tested^31^. Our result with siRNA-induced knockdown of *LARGE* expression further supports this notion. These results therefore suggest that ribitol and CDP-ribitol act prior to the synthesis of LARGE-induced biglycan repeats, but, not on the expression of DG protein itself, as evidenced by abundant low MW α-DG representing the core M3 detected by antibody AF6868 in untreated MCF7 and T47D cells.

The mechanisms for ribitol-enhanced matriglycan expression is not fully understood. Several genes are known to be essential for the LARGE-mediated synthesis of matriglycan. They include *POMGnT2* (*GTDC2*) (14,15), *B3GALNT2*(16), *FKTN*^9,17,18^, *FKRP*^9,17,18^, *TMEM5*(12) and *B4GAT1*(11). However, among these genes, only FKRP and FKTN function as ribitol-5P transferases with CDP-ribitol as the immediate donor substrate and therefore can be perceived as directly relevant. An early study by Gerin *et al*. demonstrated that ribitol treatment leads to an increase of CDP-ribitol levels in HEK293 cells, patient-derived ISPD-deficient fibroblasts and normal muscles *in vivo*^18,38^. We have recently demonstrated that ribitol treatment is able to increase the levels of CDP-ribitol substantially in cardiac and skeletal muscles^33^. In the current study, we further show that ribitol treatment increases levels of CDP-ribitol in epithelial cells. The increase in the substrate therefore likely enhances the efficiency of FKRP and FKTN function, and thereby the levels of matriglycan on α-DG. This hypothesis is supported by our study in the FKRP mutant mouse model in which ribitol treatment leads to the restoration of matriglycan in body-wide muscles without effecting *FKRP* and *LARGE* expression^33^. Since the only defect in the FKRP mutant mice is the *FKRP* gene, restoration of matriglycan in the model can be considered the result of improved activity of the mutant FKRP which is known to retain partial function^39^. We therefore hypothesize that ribitol treatment of the cancer cells increases CDP-ribitol which in turn improves efficiency of normal FKRP (confirmed by sequencing and the data is included in the Supplementary Information Fig. 6), and possibly FKTN, and thus the levels of matriglycan synthesis. This hypothesis agrees with the general concept of enzyme kinetics that variation in concentration of substrate can alter the rate of enzyme reaction before it reaches saturation. Nevertheless, this hypothesis remains to be investigated and involvement of other factors could not be excluded.

The effect of ribitol-induced enhancement of matriglycan on cancer cells *in vitro* is apparently in contrast to that of *LARGE* overexpression-induced hyperglycosylation^31^. Ribitol significantly enhanced the expression of matriglycan, but yet this enhancement is associated with neither reduced cell proliferation, nor clear alteration in cell cycle progression and the capacity of growth in Matrigel of the cancer cells. It is possible that the levels of matriglycan induced by ribitol in the cell culture remain insufficient to achieve significant inhibition as LARGE-induced hyperglycosylation which is known to be considerably higher than normal levels in any cells^31,40^. Another obvious difference is the nature of the enhanced matriglycan. LARGE overexpression enhances matriglycan through extended biglycan repeats as indicated by the significant increase in MW of α-DG when compared to the α-DG in corresponding normal tissues^31,40^. In contrast, ribitol treatment apparently only increases the matriglycan of normal sizes (Fig. 1). LARGE has also been reported to possibly act on the *O*-mannose, complex N-glycans and mucin *O*-GalNAc glycans of α-DG, or to modify glycosylation on proteins other than α-DG, thus potentially affecting cell behavior^41,42^. Furthermore, high levels of matriglycan expression have been reported in metastatic tumors of clinical samples and in cell lines such as T47D, suggesting that the effect of matriglycan on tumor cell behavior may well be cell-type specific. Thus, both levels of matriglycan and sizes of matriglycan chain on α-DG as well as cell types could be important.

In summary, our results demonstrate for the first time that ribitol and CDP-ribitol are able to enhance matriglycan in cancer cells and add another layer of potential regulation for matriglycan expression by substrates. Variable and particularly high levels of matriglycan expression in primary and metastatic cancer samples and cell lines support the notion that matriglycan is one of potentially many factors which can act on cell proliferation and cancer progression. The effect of enhanced matriglycan expression and its therapeutic value as a cancer treatment therefore could well be tumor cell type specific, or dependent on other factors yet to be defined. Additionally, the nature of enhanced matriglycan and other potential effects of individual enhancers exemplified by ribitol and LARGE over expression, could also result in varied biological consequence on cells beyond effect of laminin binding matriglycan. This implies that specific genotype and phenotypes responsive to ribitol or LARGE-induced matriglycan expression will need to be identified for potential therapeutic exploitation.

## Materials and Methods

### Tumor cell lines

The human breast cancer cell lines MCF7 (ATCC- HTB-22), T47D (ATCC-CRL2865), MDA-MB-231 (MDA-231) (ATCC-HTB-26), prostate cancer cell lines LNCaP (ATCC-CRL-1740), PC3 (ATCC-CRL-1435), Lung cancer cell line NCI-H1299 (ATCC-CRL-5803), and Cervix cancer cell line HeLa (ATCC CCL-2) were used.

MCF7 and T47D were grown in DMEM-GlutaMAX (10569, Gibco by life technologies) plus 10% fetal bovine serum (FBS 10082-147) and 10μg/ml insulin (I5500 Sigma). MDA-231 and Hela were grown in DMEM-GlutaMAX+10% FBS at 37 °C in a 10% CO_2_ incubator. LNCaP and H1299 were grown in RPMI1640 (61870) +10% FBS. PC3 was grown in F12 Nutrient Mixture (Ham)-(11765) +10% FBS at 37 °C in a 5% CO_2_ incubator. The culture media were obtained from by Life Technologies (Carlsbad).

### Ribitol, CDP-ribitol and glucose treatment

Cells of 3 × 10^5^/well 6 well plate or 2 × 10^4^/well 96 well plate seeded in triplicate in the growth medium unless specified otherwise. The following day, the medium was supplemented with ribitol (A5502 Sigma, St. Louis) or glucose (G7528 Sigma) or CDP-ribitol (Z Biotech, Aurora, CO) at a range of specific concentrations and the cells were grown in the supplement for 3 days. Plates were washed with PBS and cells were processed for analyses by FACS, immunocytochemistry and western blot.

### siRNA transfection and sample preparation

Human LARGE siRNAs (SmartPoll Accell E-011920-00-0005) were obtained from Dharmacon LLC. MCF7 and T47D cells (2 × 10^5^/well of 12 well plate) were seeded an hour before siRNA transfection. HiPerFect Transfection Reagent (QIAGEN, Germantown, MD) for adherent cell transfection with siRNA was used per manufacture instruction. Three concentrations, 1 nmol, 5 nmol and 10 nmol, were tested. Three days after transfection, cells were collected for total RNA isolation by Trizol reagent (15596026, Invitrogen, Carlsbad, California), and the isolated RNA was proceeded for measurement of LARGE mRNA by quantitative Real Time PCR Assay described below. To examine the effect of LARGE knock-down on ribitol-mediated matriglycan enhancement, ribitol at 5 mM concentration was added 5 hours after the siRNA transfection with the same 3 siRNA concentrations. Three days after transfection, the treated cells were processed for Immunocytochemistry staining for matriglycan expression with IIH6C4 (IIH6) antibody (Millipore, Temecula, CA) as described below.

### Flow cytometry

Antibody IIH6 was used to assess the level of matriglycan on α-DG. Cells were harvested by gentle cell scraper and total number counted. Cells were first blocked with 1% FBS and 1% normal goat serum in PBS for 30 minutes on ice, washed with PBS and then incubated with IIH6 at 1:100 dilution in PBS/0.1%FBS for 60 min on ice. After wash with PBS 2 times, the cells were stained with secondary antibody Alex 594 conjugated goat-anti-mouse/IgM (Life Technologies, Carlsbad, CA) at 1:100 dilution for 45 min on ice, washed and resuspended in 500 μl FACS buffer containing 1% BSA, 0.1% NaN3 in PBS and then analyzed by FACS. All experiments were done in triplicate.

### Immunocytochemistry (ICC)

Cell cultures for ICC were washed with PBS before fixation with ice-cold methanol for 10 minutes. Residual methanol was removed by washing with PBS and air dry. Cell were rehydrated prior to staining procedures with PBS and blocked with 6% bovine serum albumin (BSA), 2% normal goat serum (NGS) in PBS for 30 minutes. Primary antibody IIH6 against α-DG in 1% BSA at 1:600 dilution was incubated 4 hours at RT or overnight at 4 °C. Samples were washed three times for 10 minutes with PBS and finally incubated with Alexa Fluor 488-conjugated goat anti-mouse IgM or Alexa Fluor 594-conjugated goat anti-mouse IgM secondary antibodies (Life Technologies, Carlsbad) at 1:600 dilution. Samples stained without primary antibody were used as control (33).

### Laminin overlay assay and western blot

The level of α-DG glycosylation was assessed by subjecting 60 μg each of total cell lysates to immunoblot analysis. Cells were lysed in Triton lysis buffer containing 1% Triton X-100, 50 mM Tris pH 8, 150 mM NaCl, 1 mM EDTA, and 1x protease Inhibitor Cocktail (Sigma). After clarification of the lysates by centrifugation at 13,000 rpm for 10 min at 4 °C, protein concentration of the lysates was measured using the Bradford method (Bio-Rad Laboratories). Samples were then electrophoretically separated on a 4–15% Criterion Tris-HCI 18-well gel, (3450028, Bio-Rad Laboratories) and transferred onto supported nitrocellulose membrane. Immunoblots were probed with primary antibody IIH6C4 at 1:1000 dilution in 3% nonfat dry milk PBS. A sheep anti-α-DG core antibody, AF6868 (R&D Systems, Minneapolis, MN), was used at 1:1000 dilution. Rabbit polyclonal antibody to actin (A2066 Sigma) was used at 1:3000 dilution in 5% nonfat dry milk/1xTBS-0.05% Tween. The blots were incubated with primary antibody overnight at 4 °C. After wash, membranes were subsequently incubated with secondary antibodies of HRP-conjugated goat anti-mouse IgM (1:3000), or goat anti-rabbit IgG (1:3000) in their blocking buffer for 1 hour 30 min. Bands were detected using Western Lightning Plus-ECL NEL 104001EA (PerkinElmer) (33).

Laminin binding assay: membranes with transferred proteins were blocked at 4 °C for 1 h in laminin binding buffer (LBB: 10 mM ethanolamine, 140 mM NaCl, 1 mM MgCl_2_ and 1 mM CaCl_2_, pH7.4) containing 5% nonfat dry milk, and then probed with laminin binding with 2 μg/ml laminin-1 in LBB milk overnight at 4 °C. After wash in LBB for 10 minx3, membrane bound laminin was detected using rabbit anti-laminin at 1:1500 dilution. Finally, membrane was incubated with HRP-conjugated goat-anti-rabbit-antibody and processed for ECL detection (33).

### Cell cycle analysis

Propidium Iodide (PI) Staining method was used as described in the DNA content determination protocol from *In Living Color. Protocols in Flow Cytometry and Cell Sorting*. R.A. Diamond and S. DeMaggio (Eds.) Springer Lab Manual, 2000, p. 361. Cells were harvested by trypsinization and washed in PBS. Cell pellet was resuspended in 500 µl of cold PBS and fixed in 5 ml cold 70% ethanol by dropwise addition to cell pellet while vortexing to ensure fixation of all cells and minimize clumping. Cells were then fixed for at least 30 min on ice and spun down at 2000 rpm for 5 min. Once in 70% ethanol, cell samples were kept for up to 2 weeks. Cells were washed twice with PBS and 100 μl ribonuclease (100 μg/ml, DNase free, Sigma) solution in PBS was directly added to pellet and incubated for 5 min at room temperature. This was followed by addition of 400 μl propidium iodide (PI) staining solution containing 50 μg/ml PI, 0.1% Triton X-100 in PBS directly to the cells in RNase solution. The cells were further Incubated for 5 to 10 minutes at room temperature and then analyzed by flow cytometry.

### Cell migration

Cell migration was assayed following manufacturer protocol: Oris Cell Migration Assay-Collagen 1 Coated 96well plate, Product No: CMACC1.101, Platypus technologies, LLC. For each cell line, 100 μl of 2.5 × 10^4^ cells were dispensed into the collagen coated seeding area in triplicates. The plate was incubated at 37 °C, 10% CO_2_ with the centered cell seeding stoppers fixed in place. The cell migration into the center detection zone was recorded 2 days after removal of stoppers. The culture was maintained in growth media only or supplemented with 20 and 50 mM ribitol for 4 days. Cells were stained with Hoechst 1322. Area within the growth circle was determined using ImageJ Software (ver. 1.51 P) measuring the relative percentage of Hoechst staining to the total area. (Rasband WS. ImageJ, U.S. National Institutes of Health, Bethesda, Maryland, USA, https://imagej.nih.gov/ij).

### Matrigel cell culture

Matrigel (Corning, VWR Atlanta, GA) was prepared according to manufacturer’s recommendation and used at 0.4 mg/ml. A total of 1 × 10^5^ cells in 100 μl of the prepared Matrigel solution was evenly distributed onto DMEM growth media-equilibrated 12 mm 3 µm pore size Millicell-PCF membrane culture plate inserts (Millipore) which was placed in 24-well polystyrene cell-culture-treated plates. Plated Matrigel cell suspension was allowed to set at 37 ° C in a humidified 10% CO_2_ circulating incubator, before washing away unincorporated cells from the top of the Matrigel surface. The culture was allowed to grow 14 days suspended in media with or without ribitol (10 mM) which was replenished every 96 hours (4 days) for the duration of the experiment. Finally, the cultures were washed by gentle submersion in changes of warmed PBS and then fixed with 4% PFA for 90 minutes. Fixed cultures were visualized by 0.01% Cresyl Violet staining solution for 30 minutes followed by soaking in changes of PBS until excess dye was completely removed. Photomicrographs of the entire Matrigel suspension in each Millicell well insert were captured at calibrated distance with an Olympus SZ61 stereoscope with illumination from an Olympus Highlight 3100, using Lumenera INFINITY 2 camera (Lumenera Corp). An over-under threshold was applied to the resulting images to define colony boundaries, which were used for colony counting and particle analysis function using ImageJ ver.1.51 P software (Rasband WS. ImageJ, U.S. National Institutes of Health, Bethesda, Maryland, USA, https://imagej.nih.gov/ij/). All measures were performed on 7 replicates, with scale determined from 1 mm grid images captured at the same focal length and magnification as the images analyzed.

Cells cultured in the Matrigel for 7 days were also collected and embedded in OCT compound (Tissue-Tek, Sakura Finetek, CA, USA), snap-frozen and cut into sections of 6 micrometers in thickness. The sections were then stained with IIH6 antibody by immunocytochemistry as described above.

### Quantitative real time PCR assay

RNA was extracted from cells using TRIzol (Invitrogen) following the supplied protocol. Final RNA pellet was re-suspended in RNAse-nuclease free water. RNA concentration was determined using Nanodrop 2000c. 500 ng of RNA was subsequently converted to cDNA using the High-Capacity RNA-to-cDNA Kit (Applied Biosystems) following the supplied protocol. cDNA was then used for quantitative real-time PCR using Taqman assays for human *FKRP* (Hs00225601_m1), *LARGE* (Hs00893935_m1), *LARGEII* (Hs00697621_g1), *ISPD* (Hs00417152_m1), and *FKTN* (Hs01121842_m1) with primer limited *ACTB* (Hs99999903_m1) as the internal control and TaqMan Universal Master Mix II, with UNG (Life Technologies). Real time PCR was run on the BioRad CFX96 Touch Real-Time PCR Detection System (BioRad) following the standard real time PCR conditions suggested for Taqman assays. Results of all expression levels were calculated as the 2^−∆∆Ct^ and compared to untreated control cells (33).

### Metabolite extraction from cells and LC-MS/MS analysis

Ribitol was purchased from Sigma (A5502). Ribitol-5-phosphate and CDP-ribitol were synthesized by Z Biotech (Aurora, CO). MCF7 cells were incubated with or without10 mM ribitol in growth medium for 3 days, harvested by trypsinization, and washed with PBS three times. Total cells of 10^5^ and 10^6^ each were analyzed for three metabolites: ribitol, ribitol 5 phosphate (rib-5P) and CDP-ribitol. Samples were blinded and then subjected to the following procedures. Cells were homogenized with 400 μl of MeOH:Acetonitrile (ACN) (1:1) and then centrifugated for 5 min at 10,000 rpm. The supernatants were removed, transferred to individual wells of 96-well plate and analyzed by LC/MS-MS performed by Z Biotech. An Applied Biosystems Sciex 4000 (Applied Biosystems; Foster City, CA) equipped with a Shimadzu HPLC (Shimadzu Scientific Instruments, Inc. Columbia, MD) and Auto-sampler (LEAP Technologies; Carrboro, NC) were used to detect ribitol, ribitol-5P and CDP-ribitol. The analysis of metabolites was performed by Z Biotech, LLC (Aurora, CO) and described previously (33).

### Statistical analysis

All data are expressed as mean ± SEM unless stated otherwise. Statistical analyses were performed with GraphPad Prism version 7.01 for Windows (GraphPad Software). Individual means were compared using unpaired *t* tests. Differences were considered to be statistically significant at *p* ≤ 0.05 (*).

## Supplementary information

Supplementary Information.

## Data Availability

All data generated/analyzed in this study are included in this article or in the Supplementary Information files and can be provided upon request.
