# Supplementary material for: Ribitol enhances matriglycan of α-dystroglycan in breast cancer cells without affecting cell growth
Source: Sci Rep. 2020 Mar 18;10:4935. doi: 10.1038/s41598-020-61747-z (PMC7080755; doi:10.1038/s41598-020-61747-z)
Supplement: Supplementary file 1 — Supplementary Information. [file 41598_2020_61747_MOESM1_ESM.pdf]

Title: **Ribitol enhances matriglycan of α-dystroglycan in breast cancer cells without affecting cell growth**

Pei Juan Lu, Jason D. Tucker, Elizabeth K. Branch, Fei Guo, Anthony R. Blaeser, Qi Long Lu

Supplemental Information (SI)

Supplemental Information (SI)

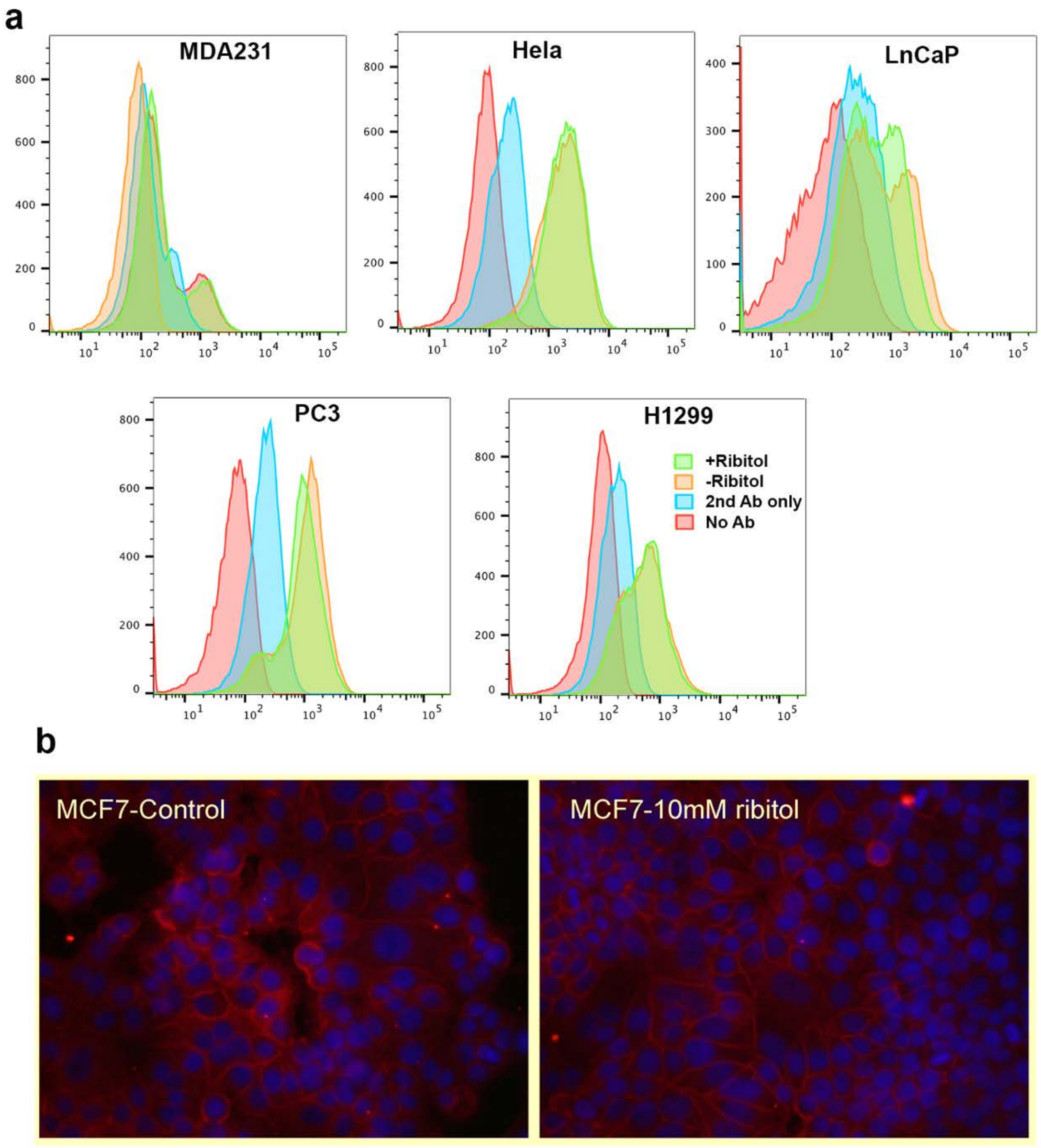

SI Figure 1. (a) Detection of F-α-DG in cancer cell lines treated with 10 mM ribitol by FACS. +Ribitol, ribitol-treated cells stained with IIH6 antibody and fluorescence labelled secondary antibody; control, untreated cells stained with IIH6 and fluorescence labelled secondary antibody; 2nd Ab only, ribitol-treated cells stained with secondary antibody only; No Ab, ribitol-treated cells without primary and secondary antibody. X axis, fluorescence intensity; Y axis, number of cells. (b) Detection of α-DG with antibody AF6868. No clear difference in signal intensity and distribution is observed between the control (untreated) and ribitol treated cells.

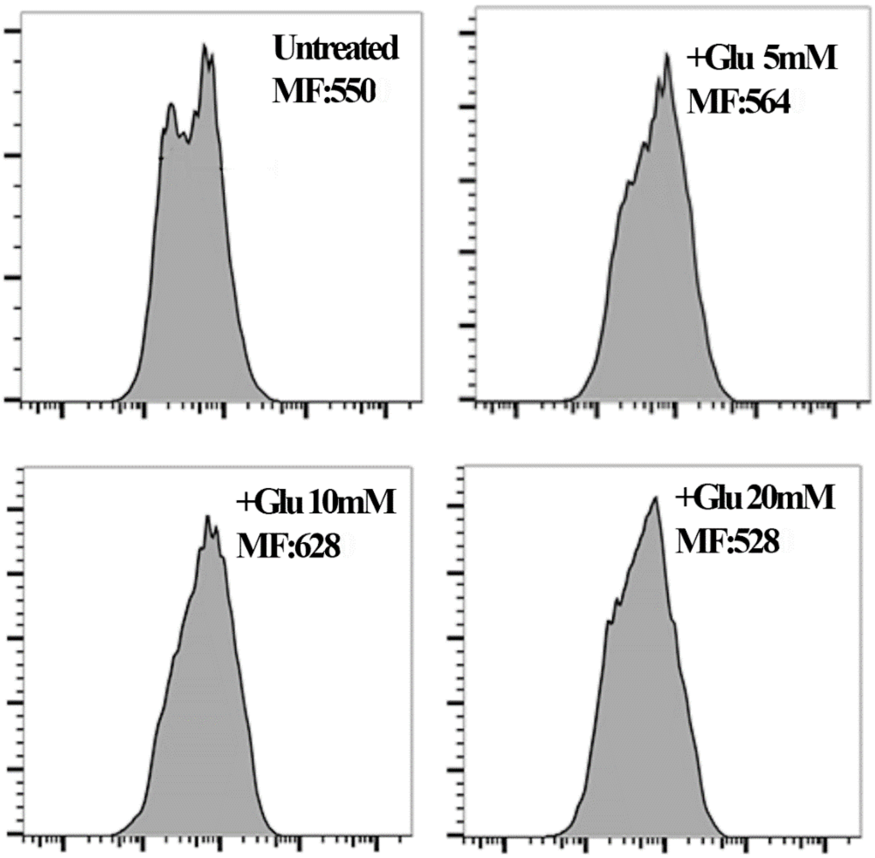

SI Figure 2. Detection of F-α-DG by FACS analysis with the antibody IIH6 in the MCF7 cells treated with D-glucose. Untreated control cells. +Glu, D-glucose-treated cells. MF, Median fluorescence reading. X axis, fluorescence intensity; Y axis, number of cells.

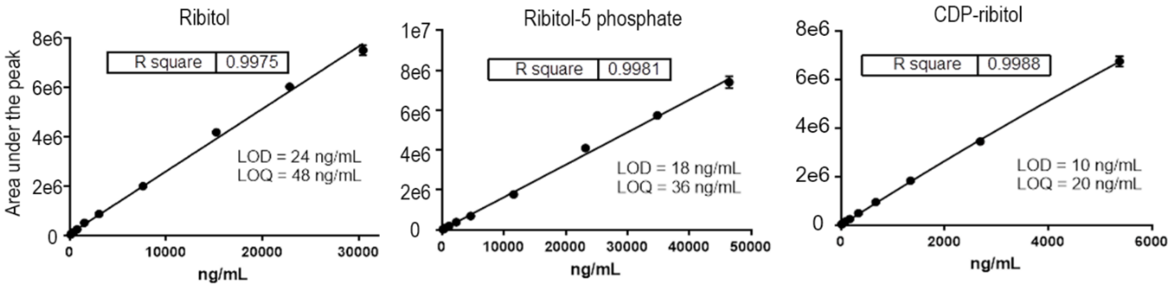

SI Figure 3. Standard curves for detection of ribitol, ribitol-5-Phosphate and CDP-ribitol.

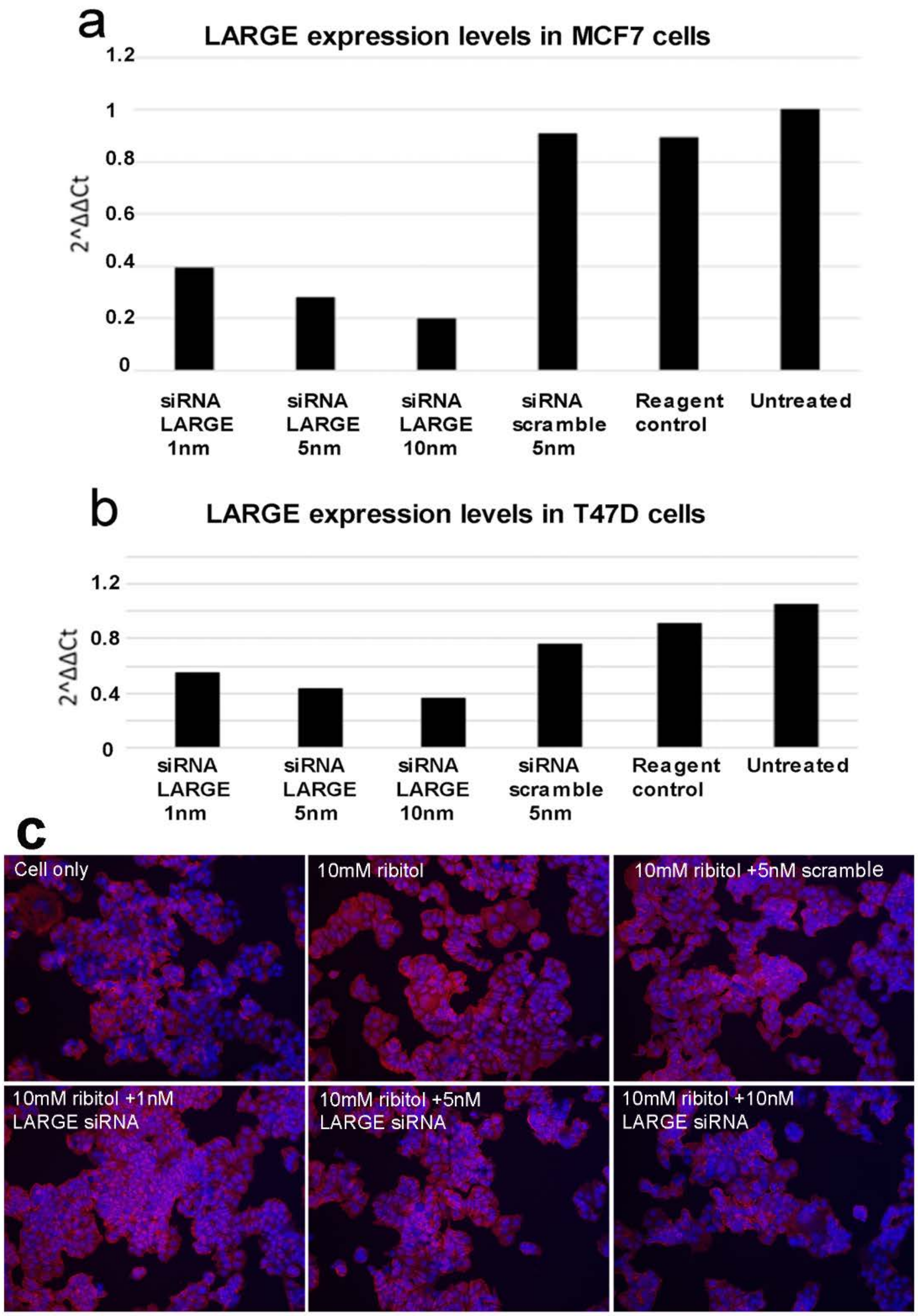

SI Figure 4. Quantitative real time RT PCR detection of *LARGE1* with siRNA treatment. Relative expression of LARGE mRNA in LARGE siRNA treated MCF7 cells (a) and T47D cells (b). Untreated controls: Scramble siRNA, Reagent control: HiPerFect Tranfection Reagent (QIAGEN) , Untreated: MCF7 cells only. Triplicate cultures were combined for the qRTPCR. Cells were treated with 1, 5 or 10nM LARGE-siRNA or Scramble-siRNA. Relative expression of *LARGE* was normalized to untreated control cells. (c) Immunocytochemistry with IIH6 antibody after the T47D cells were treated 10 mM ribitol for 3 days. A limited reduction in positive signal was observed with the increase in dose of siRNA LARGE from 1 nM to 10 nM. Red fluorescence is the signal for IIH6 and blue is DAPI staining for nuclei.

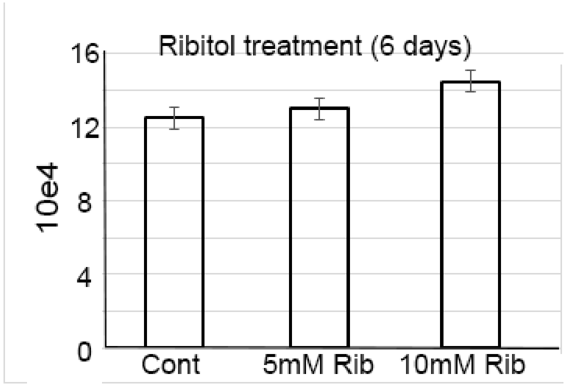

SI Figure 5. Cell number of MCF7 in growth medium with ribitol for 6 days. Cont, control cells without ribitol; 5mM Rib and 10mM Rib, cells treated with 5 mM and 10 mM ribitol. (n=3, P>0.05).

```
        10        20        30        40        50        60        70        80        90
      CCTCTGACTACCATTTCTAAGGATGCCCCGGAGGCCCAGCTAGCCCCAGACTTCGGCCCCATGCGGCTCACCCGCTGCCAGGCTGCCCTGGCGGC
3 ←                        GATGCCCCGGAGGCCCAGCTAGCCCCAGACTTCGGCCCCATGCGGCTCACCCGCTGCCAGGCTGCCCTGGCGGC

       100       110       120       130       140       150       160       170       180       190
      CGCCATCACCCTCAACCTTCTGGTCCTCTTCTATGTCTCGTGGCTGCAGCACCAGCCTAGGAATTCCCGGGCCCGGGGGCCCCGTCGTGCCTCTG
1 →                                                                                           GCCTCTG
3 ←   CGCCATCACCCTCAACCTTCTGGTCCTCTTCTATGTCTCGTGGCTGCAGCACCAGCCTAGGAATTCCCGGGCCCGGGGGCCCCGTCGTGCCTCTG

       200       210       220       230       240       250       260       270       280
      CTGCCGGCCCCCGTGTCACCGTCCTGGTGCGGGAGTTCGAGGCATTTGACAACGCGGTGCCCGAGCTGGTAGACTCCTTCCTGCAGCAAGACCCA
1 →   CTGCCGGCCCCCGTGTCACCGTCCTGGTGCGGGAGTTCGAGGCATTTGACAACGCGGTGCCCGAGCTGGTAGACTCCTTCCTGCAGCAAGACCCA
3 ←   CTGCCGGCCCCCGTGTCACCGTCCTGGTGCGGGAGTTCGAGGCATTTGACAACGCGGTGCCCGAGCTGGTAGACTCCTTCCTGCAGCAAGACCCA

       290       300       310       320       330       340       350       360       370       380
      GCCCAGCCCGTGGTGGTGGCAGCCGACACGCTCCCCTACCCGCCCCTGGCCCTGCCCCGCATCCCCAACGTGCGTCTGGCGCTGCTCCAGCCCGC
1 →   GCCCAGCCCGTGGTGGTGGCAGCCGACACGCTCCCCTACCCGCCCCTGGCCCTGCCCCGCATCCCCAACGTGCGTCTGGCGCTGCTCCAGCCCGC
3 ←   GCCCAGCCCGTGGTGGTGGCAGCCGACACGCTCCCCTACCCGCCCCTGGCCCTGCCCCGCATCCCCAACGTGCGTCTGGCGCTGCTCCAGCCCGC

       390       400       410       420       430       440       450       460       470
      CCTGGACCGGCCAGCCGCAGCCTCGCGCCCGGAGACCTACGTGGCCACCGAGTTTGTGGCCCTAGTACCTGATGGGGCGCGGGCTGAGGCACCTG
1 →   CCTGGACCGGCCAGCCGCAGCCTCGCGCCCGGAGACCTACGTGGCCACCGAGTTTGTGGCCCTAGTACCTGATGGGGCGCGGGCTGAGGCACCTG
3 ←   CCTGGACCGGCCAGCCGCAGCCTCGCGCCCGGAGACCTACGTGGCCACCGAGTTTGTGGCCCTAGTACCTGATGGGGCGCGGGCTGAGGCACCTG

       480       490       500       510       520       530       540       550       560       570
      GCCTGCTGGAGCGCATGGTGGAGGCGCTCCGCGCAGGAAGCGCACGTCTGGTGGCCGCCCCGGTTGCCACGGCCAACCCTGCCAGGTGCCTGGCC
1 →   GCCTGCTGGAGCGCATGGTGGAGGCGCTCCGCGCAGGAAGCGCACGTCTGGTGGCCGCCCCGGTTGCCACGGCCAACCCTGCCAGGTGCCTGGCC
3 ←   GCC

       580       590       600       610       620       630       640       650       660
      CTGAACGTCAGCCTGCGAGAGTGGACCGCCCGCTATGGCGCAGCCCCCGCCGCGCCCCGCTGCGACGCCCTGGACGGAGATGCTGTGGTGCTCCT
1 →   CTGAACGTCAGCCTGCGAGAGTGGACCGCCCGCTATGGCGCAGCCCCCGCCGCGCCCCGCTGCGACGCCCTGGACGGAGATGCTGTGGTGCTCCT

       670       680       690       700       710       720       730       740       750       760
      GCGCGCCCGCGACCTCTTCAACCTCTCGGCGCCCCTGGCCCGGCCGGTGGGCACCAGCCTCTTTCTGCAGACCGCCCTTCGCGGCTGGGCGGTGC
1 →   GCGCGCCCGCGACCTCTTCAACCTCTCGGCGCCCCTGGCCCGGCCGGTGGGCACCAGCCTCTTTCTGCAGACCGCCCTTCGCGGCTGGGCGGTGC

       770       780       790       800       810       820       830       840       850
      AGCTGCTGGACTTGACCTTCGCCGCGGCGCGCCAGCCCCCGCTGGCCACGGCCCACGCGCGCTGGAAGGCTGAGCGCGAGGGACGCGCTCGGCGG
1 →   AGCTGCTGGACTTGACCTTCGCCGCGGCGCGCCAGCCCCCGCTGGCCACGGCCCACGCGCGCTGGAAGGCTGAGCGCGAGGGACGCGCTCGGCGG

       860       870       880       890       900       910       920       930       940       950
      GCGGCGCTGCTCCGCGCGCTGGGCATCCGCCTAGTGAGCTGGGAAGGCGGGCGGCTGGAGTGGTTCGGCTGCAACAAGGAGACCACGCGCTGCTT
1 →   GCGGCGCTGCTCCGCGCGCTGGGCATCCGCCTAGTGAGCTGGGAAGGCGGGCGGCTGGAGTGGTTCGGCTGCAACAAGGAGACCACGCGCTGCTT
2 →                                                                               AGGAGACCACGCGCTGCTT

       960       970       980       990      1000      1010      1020      1030      1040
      CGGAACCGTGGTGGGCGACACGCCCGCCTACCTCTACGAGGAGCGCTGGACGCCCCCCTGCTGCCTGCGCGCGCTGCGCGAGACCGCCCCGCTATG
1 →   CGGAACCGTGGTGGGCGACACGCCCGCCTACCTCTACGAGGAGCGCTGGACGCCCCCCTGCTGCCTGCGCGCGCTGCGCGAGACCGCCCCGCTATG
2 →   CGGAACCGTGGTGGGCGACACGCCCGCCTACCTCTACGAGGAGCGCTGGACGCCCCCCTGCTGCCTGCGCGCGCTGCGCGAGACCGCCCCGCTATG
```

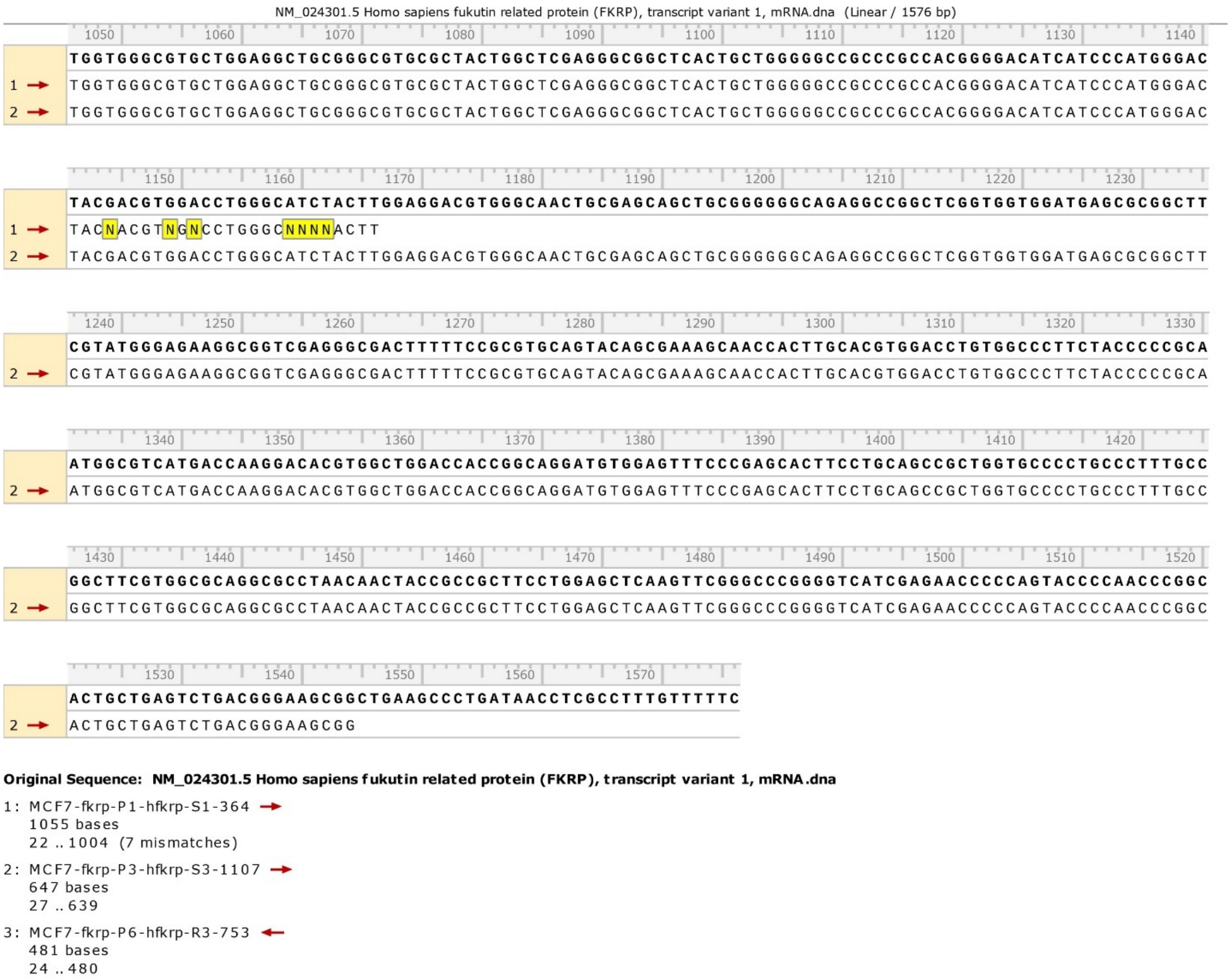

SI Figure 6. DNA sequencing for FKRP gene. Full-length coding sequence of the human fkrp was directly amplified by PCR from genomic DNA isolated from cultured MCF7 cells with the following primers: hfkrp-G2-F, GATGCCCCGGAGGCCCAGCT and hfkrp-R2, AGTCTGACGGGAAGCGG. The PCR reaction included 0.5 ug DNA, 200 uM dNTPs, 400 nM primer each, 3.5% DMSO, 1xPCR buffer and 5 units of Taq DNA polymerase (Promega M166, Madison WI USA). The 1523bp product was isolated using QIAquick Gel Extraction Kit (QIAGEN). The purified DNA was sequenced by Genewiz DNA Sequencing Services, South Plainfield NJ 07080 USA with the following sequencing primers: P1-hfkrp-S1-364, CTGCAGCACCAGCCTAGGA; P3-hfkrp-S3-1107, CCGCCTAGTGAGCTGGGAA and P6-hfkrp-R3-753, CACCAGACGTGCGCTTCCT. The sequences are aligned with the normal FKRP mRNA sequence (original sequence, top lines).

MS/MS Fragments		538 --> 324		153 --> 98.8		233 --> 98.8	538 --> 324
Cells		CDP-Ribitol*	SD	Ribitol *	SD	Ribitol-Phosphate*	SD
1	MCF7-rib10^5	0.2	0.1	6.3	1.3	2.7	3.8
2	MCF7-rib10^6	2.7	0.1	30.2	3.3	16.4	1.9
3	MCF7+rib-10^5	2.6	0.2	243.6	7.7	29.6	1.5
4	MCF7+rib-10^6	27.1	0.1	1532.9	55.0	168.9	2.3

Supplementary Table 1. Measurement of ribitol, ribitol-5-Phosphate and CDP-ribitol by the LC/MS/MS. Amount (ng) of the substances is determined from total 1e5 and 1e6 cells of the untreated (-rib) and 10 mM ribitol-treated (+rib) samples. The amount of metabolites represents total amount of metabolite estimated from the Standard curves. * ng

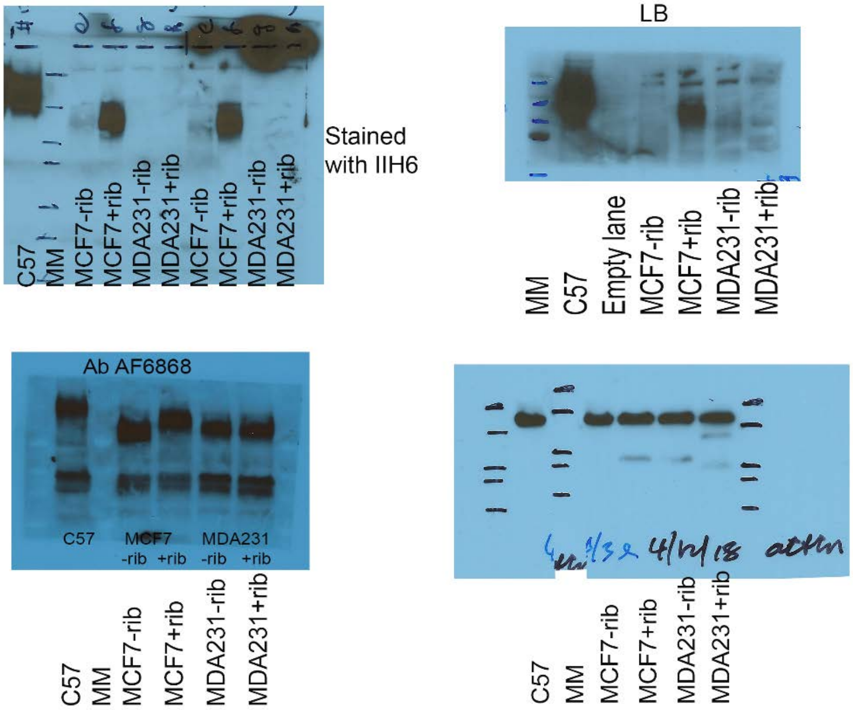

Uncropped western blots of the Figure 1c. Samples from MDA231 and MCF7 probed with IIH6 and AF6868 are duplicates.

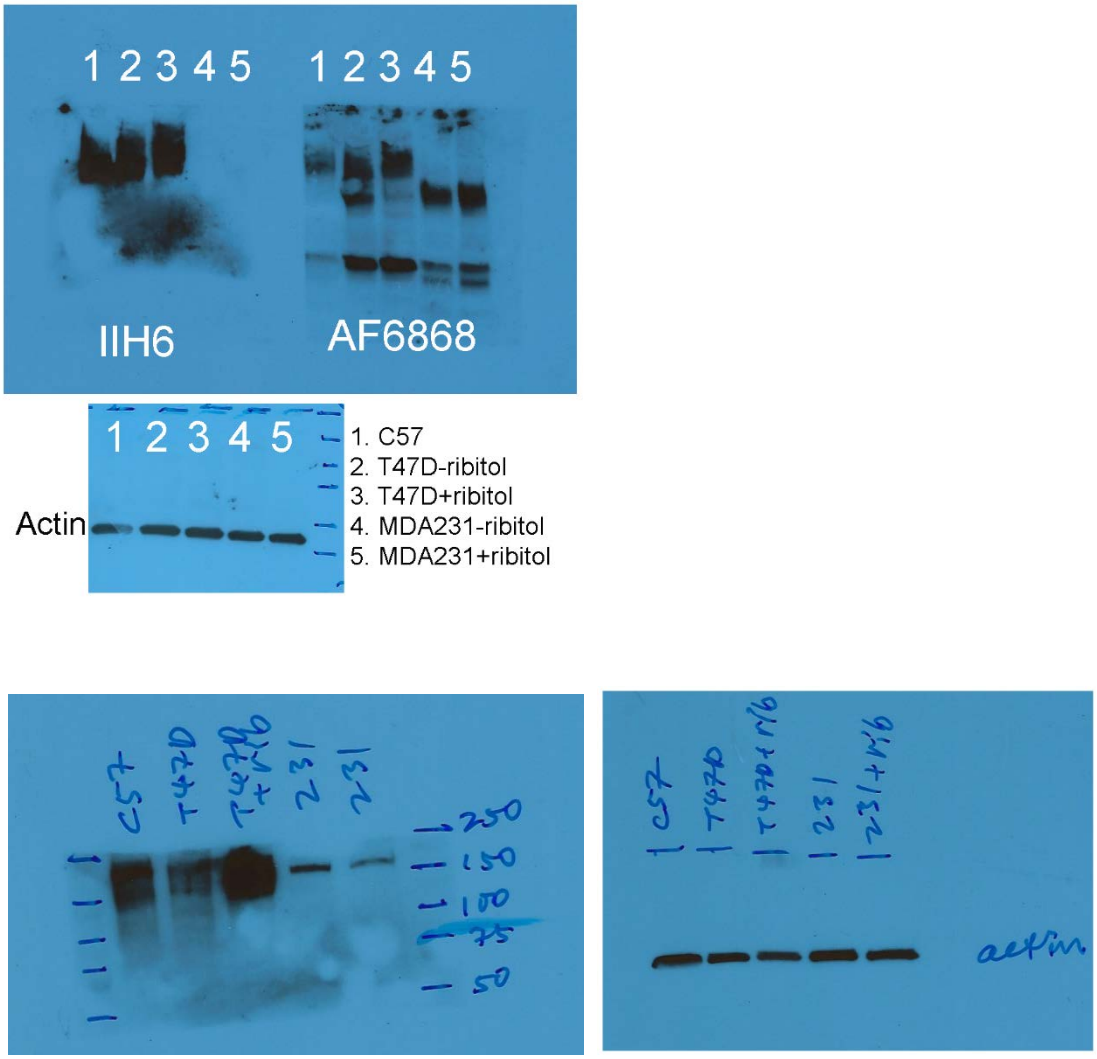

Uncropped western blots of the Figure 3a.
